# Variations in volatile flavour compounds in *Crataegi* fructus roasting revealed by E-nose and HS-GC-MS

**DOI:** 10.3389/fnut.2022.1035623

**Published:** 2023-01-25

**Authors:** Chenghao Fei, Qianqian Xue, Wenjing Li, Yan Xu, Liyan Mou, Weidong Li, Tulin Lu, Wu Yin, Lin Li, Fangzhou Yin

**Affiliations:** ^1^School of Pharmacy, Nanjing University of Chinese Medicine, Nanjing, China; ^2^State Key Laboratory of Pharmaceutical Biotechnology, College of Life Sciences, Nanjing University, Nanjing, China

**Keywords:** *Crataegi* fructus, E-nose, HS-GC-MS, roasting, volatile flavour compounds

## Abstract

**Introduction:**

*Crataegi* fructus (CF) is an edible and medicinal functional food used worldwide that enhances digestion if consumed in the roasted form. The odour of CF, as a measure of processing degree during roasting, significantly changes. However, the changes remain unclear, but are worth exploring.

**Methods:**

Herein, the variations in volatile flavour compounds due to CF roasting were investigated using an electronic nose (E-nose) and headspace gas chromatography–mass spectrometry (HS-GC-MS).

**Results:**

A total of 54 components were identified by GC-MS. Aldehydes, ketones, esters, and furans showed the most significant changes. The Maillard reaction, Strecker degradation, and fatty acid oxidation and degradation are the main reactions that occur during roasting. The results of grey relational analysis (GRA) showed that 25 volatile compounds were closely related to odour (*r* > 0.9). Finally, 9 volatile components [relative odour activity value, (ROAV) ≥ 1] were confirmed as key substances causing odour changes.

**Discussion:**

This study not only achieves the objectification of odour evaluation during food processing, but also verifies the applicability and similarity of the E-nose and HS-GC-MS.

## 1. Introduction

Food processing converts normally inedible raw materials into edible, safe, and nutritious foods and plays an important role in food preservation and biotransformation (1). Food processing technology has evolved over thousands of years, from roasting meat over the fire approximately 1.8 million years ago, to current methods including cooking, preservation using heat, pickling, fermenting, freezing, and drying (2). In particular, roasting is widely used processing methods, wherein the temperature and time are critical factors (3). Many foods are processed by roasting, including coffee, tea, peanuts, and barley (4, 5). Food processing by various methods induces changes in physical properties and is often accompanied by biochemical reactions (6). The complexity of food processing methods introduces new challenges for food quality evaluation.

Food flavours generally change during processing and the composition of volatile components is key to food flavour, actively affecting its overall evaluation (7). As an example, volatile components of fish (ammonia, indole, histamine, trimethylamine, and ammonia sulphide) can be used as indicators for detecting changes in freshness (8). It has been reported that the odour is directly related to the presence of volatile substances (9, 10). However, the judgment of food odour depends on human olfactory sensation, which is subjective and affected by the environment and the physical condition of the human, resulting in poor repeatability (11). With continuous technological developments, precise analytical instruments are gradually being developed for the study of food odour. The electronic nose (E-nose) is an odour detection technology that simulates the human olfactory system and provides low-cost and rapid sensory information for process monitoring and quality control during food production (12). Advantages of the E-nose include its rapid, sensitive, and non-destructive sample identification that objectively reflects the tested samples. Although the overall information regarding volatile flavour substances can be analysed by the E-nose, specific information on volatile components cannot be obtained. Headspace gas chromatography-mass spectrometry (HS-GC-MS) is commonly used for qualitative and quantitative analysis of volatile components (13, 14). It integrates the safety, accuracy, and high separation ability of GC with the superiority of MS for substance identification. Compared to the E-nose, GC-MS can provide detailed and accurate information regarding volatile flavour components (15). However, online sample monitoring is difficult due to high cost and long cycle for analysis. The E-nose and GC-MS can be used for quality control, fresh grading, and authenticity determination for fruits, vegetables, meat, cereals, beverages, and other products (16, 17). Although the combined use of the E-nose and GC-MS has been reported, odour characterisation by both methods is relatively independent, and their internal correlation has not been reported, meriting further exploration.

*Crataegi* fructus (CF) is harvested from plants of the genus *Crataegus*, belonging to the family Rosaceae. It is cultivated globally in regions including Asia, Europe, and North America (18). Previous studies have shown that CF has a variety of physiological functions, including digestion promotion, reducing blood lipid levels, and antibacterial activity (19, 20). CF has long been used as a traditional dual-purpose material for medicine and functional foods worldwide. Generally, fresh CF can be eaten, but it is often sliced and dried for preservation. In China, dried CF is further roasted to prevent gastrointestinal irritation caused by acidity, with three different processed products formed by roasting (21). Chao *Crataegi* fructus (CCF), whose fruity aroma decreases after roasting CF for a period of time, can promote digestion. Jiao *Crataegi* fructus (JCF), whose roasting time is longer than that for CCF and has a burnt aroma, promotes digestion and treats diarrhoea. Tan *Crataegi* fructus (TCF), whose roasting time is the longest and exhibits a charcoal-like odour, promotes haemostasis and treats diarrhoea. Therefore, during roasting, CF odours significantly change, and its sour flavour gradually diminishes, while the coke odour is enhanced. The odour changes, which are considered an intrinsic property of the fruit, indicates a change in its internal components. Previously, we established an odour detection method for CF based on the E-nose and identified differently processed CFs for the first time (22). Nevertheless, it only focused on the qualitative odour of the final products, as most of the related literature, and the detailed changes in volatile flavour substances remain unknown.

Hence, to comprehensively, reliably, and scientifically identify odour changes in CF during roasting, the E-nose and HS-GC-MS were used herein. Based on grey relational analysis (GRA) and the relative odour activity value (ROAV), a method that tracks and detects changes in volatile flavour compounds during CF roasting, was established. Taken together, these methods comprehensively and objectively reflect the changes in volatile flavour substances. The entire experimental flowchart is shown in Supplementary Figure 1.

## 2. Materials and methods

### 2.1. Sample collection and preparation

The dried CF slices were collected from Shandong of China, and the sample was roasted using CGD-750 drum roaster (Hangzhou Haishan Pharmaceutical Equipment Co., Ltd., Zhejiang, China). After the drum roaster was preheated to 200°C, the CF pieces were put into the drum roaster and roasted for 24 min. Samples were taken every 2 min. During roasting, the temperature of CF was controlled below 200°C, as monitored by infrared thermometer. Finally, 13 samples, including the original sample (S1) and 12 roasted samples (S2–S13) were obtained for further analysis. The profile of S1–13 sample are shown in Supplementary Figure 2. All the samples were pulverized and sieved (50 mesh), then the powder of the samples was sealed before analysis.

### 2.2. E-nose analysis

The E-nose analyses were conducted using a commercial Fox-4000 electronic nose (Alpha M.O.S., Toulouse, France). The structure of E-nose consists of HS-100 autosampler, sensor array units and pattern recognition system (Alpha Soft V11.0). The sensor array is composed of 18 metal oxide semiconductors as follows: LY2/LG, LY2/G, LY2/AA, LY2/GH, LY2/gCT, LY2/gCT, T30/1, P10/1, P10/2, P40/1, T70/2, PA/2, P30/1, P40/2, P30/2, T40/2, T40/1, and TA/2. The characteristics of sensors are described in Supplementary Table 1.

E-nose analysis was carried out by the method as we previously described (22). The CF samples (0.5 g) were accurately weighed and placed in 10-ml vials. Each sample was incubated at a temperature of 55°C for 600 s, then 1200 μl of the headspace air was injected into the testing chamber. The response curves were generated based on the response values acquired from 18 sensors within 120 s. Each sample was analysed in triplicate.

### 2.3. HS-GC-MS analysis

The GC-MS analysis was performed on an Agilent GC 7890B-MS 7000C (Agilent Technologies, Palo Alto, CA, USA) coupled with a 7697A headspace sampler. The sample (1.5 g) was put into 10-ml headspace vials for detection. The standard of C7–C30 saturated alkanes was provided by Sigma-Aldrich (St. Louis, MO, USA). The GC-MS analysis was referred to the published protocol with minor modifications (23).

(1) HS conditions: Equilibrium temperature and time were set at 80°C and 30 min, respectively. Loop temperature and transmission line were set at 95°C and 110°C, respectively. Oscillation frequency was set at 250 times/min. Filling pressure was set at 15 psi. Pressurization time and injection time was set at 0.1 and 0.5 min, respectively. The flow rate was maintained at 1.00 ml/min. The injection volume was 1,000 μl.

(2) GC conditions: The compounds were separated on a HP-INNOWAX capillary column (30 m × 250 μm, 0.25 μm). The injection port temperature was at 220°C in the split mode (5:1). Gasification chamber temperature was kept at 280°C. The oven temperature was programmed as follows: initial temperature was set at 40°C, ramped up to 65°C at 5°C/min, ramped up to 90°C at 10°C/min, ramped up to 110°C at 2°C/min, maintained isothermal for 10 min, then ramped up to 165°C at 5°C/min, ramped up to 260°C at 10°C/min, maintained isothermal for 5 min.

(3) MS conditions: EI source was selected. The ionization temperature was set at 230°C and mass spectra were obtained by electron energy at 70 eV. Temperature of quadrupole and detector interface were 270 and 150°C, respectively. Multiplier voltage was set at 1557.3 V and mass scan range was 45–500 amu.

The mass spectra of volatiles detected from samples were compared with those from NIST14 (National Institute of Standards and Technology, Gaithersburg, MD, USA). Based on the MS match factor (similarity > 750) and retention index (RI), each volatile compound was identified. Each sample was analysed in triplicate.

Relative odour activity value was calculated to measure the contribution of each volatile component to the aroma profile according to the method as previously described (24). The equation is as follows:


$$ROAV_{i}=100\times \frac{C_{i}}{C_{max}}\times \frac{T_{max}}{T_{i}}$$

where *C*_*i*_ and *T*_*i*_ indicate relative percentage content of each volatile component and the corresponding odour threshold (OT), respectively. *C*_*max*_ and *T*_*max*_ indicate relative percentage content of the volatile component that contributed the most to the flavor and the corresponding OT, respectively. The compounds with 0.1 ≤ ROAV < 1 modified the aroma profile, while the compounds with ROAV ≥ 1 were regarded as the key volatile components.

### 2.4. Statistical analysis

Principal component analysis (PCA) was performed with SIMCA-P 14.1 (Umetrics AB, Umea, Sweden). The heatmap was conducted using TBtools.^1^ Gray relational analysis was performed on DPS 7.05 software (Hangzhou Ruifeng Information Technology Co., Ltd., Zhejiang, China).

The basic idea of GRA is to investigate the correlation between two sets of variations (24). In this study, GRA was applied to find out the relationships between E-nose sensor responses of samples and their corresponding peak areas of volatile compounds gained by HS-GC-MS at different time-points during the processing. The specific method is as follows: Set *A*_*i*_ = {*a*_*i*_ (*k*)| *k* = 1, 2,…, *n*} = (*a*_*i*_ (1), *a*_*i*_ (2),…, *a*_*i*_ (*n*)) as the sequence of system behaviour characteristics. Set *P*_*i*_ = {*p*_*i*_ (*k*)| *k* = 1, 2,…, *n*} = (*p*_*i*_ (1), *p*_*i*_ (2),…, *p*_*i*_ (*n*)) as the sequence of system associated factors. In this study, *A*_*i*_ and *P*_*i*_ represents the data from E-nose and GC-MS, respectively. And *k* refers to the different time-point. Then, the correlation coefficient (ξ) is defined as follows:


$$\begin{array}{l} \xi \left(a_{i}\left(k\right),p_{i}\left(k\right)\right)=\\ \;\;\;\;\;\;\;\;\;\;\;\frac{\begin{array}{l} \underset{i}{\min}\,\underset{k}{\min}\left|a_{i}\left(k\right)-p_{i}\left(k\right)\right|+\rho \underset{i}{\max}\,\underset{k}{\max}\\ \;\;\;\;\;\;\;\;\;\;\;\;\left|a_{i}\left(k\right)-p_{i}\left(k\right)\right| \end{array}}{\begin{array}{l} \left|a_{i}\left(k\right)-p_{i}\left(k\right)\right|+\rho \underset{i}{\max}\,\underset{k}{\max}\\ \;\;\;\;\;\;\;\;\left|a_{i}\left(k\right)-p_{i}\left(k\right)\right| \end{array}} \end{array}$$

*ρ* is the distinctive coefficient lying between 0 and 1, and it is generally set as 0.5.

The grey correlation degree (*r*_*i*_) is the arithmetic mean of the correlation coefficient at different time-points, and is formulated as follows:


$$r_{i}=\xi \left(A_{i},P_{i}\right)=\frac{1}{n}\sum_{k=1}^{n}\xi \left(a_{i}\left(k\right),p_{i}\left(k\right)\right)$$

The higher the value of *r*_*i*_ is, the closer the sequence of associate factors to the behaviour characteristics would be.

## 3. Results and discussion

### 3.1. Sensory evaluation of roasted CF

With extended heating time and increased CF temperature, the fruity aroma of CF gradually diminished, and a burnt aroma became increasingly dominant, resulting in only a charcoal-like odour remained. Based on colour and odour judgment by experienced personnel, among the 13 obtained samples, S4 and S5 (sample temperature of approximately 100°C after roasting for 6 min) were determined to be CCF, S7 and S8 (sample temperature of approximately 130°C after roasting for 12 min) were assigned to JCF, and S13 (sample temperature of approximately 170°C after roasting for 24 min) was determined to be TCF. Corresponding images of the products are shown in Supplementary Figure 2.

### 3.2. E-nose analysis

**FIGURE 1:**
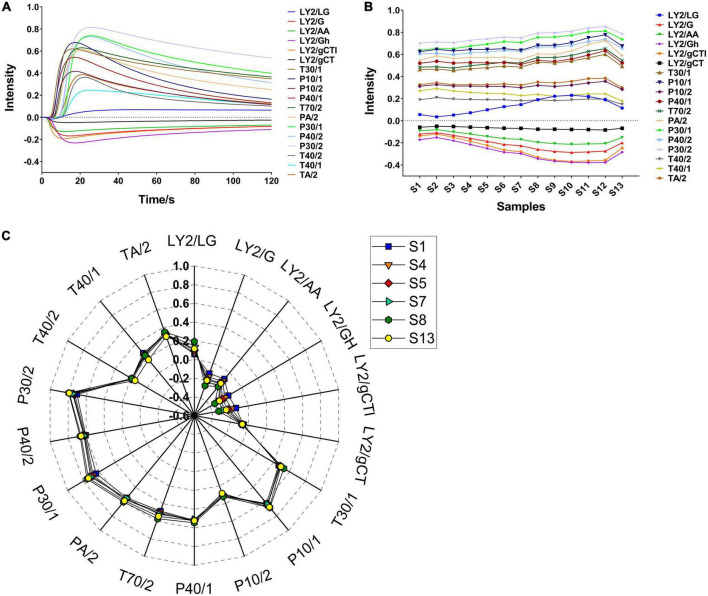
Typical plots of E-nose in CF roasting process. **(A)** Sensor intensity curves in S1; **(B)** the change of the maximum response value of all samples; **(C)** radar fingerprint chart of maximum sensor response on typical samples.

The volatile flavour characteristics of CF were comprehensively analysed using the E-nose. The sensor responses were influenced by the volatile component composition and their proportions. Representative E-nose sensor-intensity curves are shown in Figure 1A. Within 120 s, the sensor response values of LY2/G, LY2/AA, LY2/GH, LY2/gCTl, and LY2/gCT were negative due to gas oxidation on those sensors being the dominant reaction over reduction. In contrast, the response of the other sensors were positive because the gas reduction was dominant. Typically, a minimum relative standard deviation can be established for the peak or valley data in the same sample curve, allowing a maximum response value for each sensor to be extracted for further analysis. As shown in Figure 1B, the maximum response of most sensors when exposed to CF samples during roasting changed similarly, i.e., they initially increased and subsequently decreased. The highest response usually occurred at S10–S12. Notably, T40/1 and T40/2 were the only two sensors for which the maximum response decreased during this period. A total of 8 sensors showed higher response values (> 0.4), indicating higher sensitivity to the CF odour. In addition, as shown in Figure 1C, significant differences were observed for S1, S4–S5, S7–S8, and S13, indicating a noticeable alteration in the volatile compounds in CF during roasting. These differences can be attributed to the formation of novel or changes in existing volatile concentrations over time.

The above results suggest that the total concentration of volatile flavour substances during CF roasting initially increased and subsequently decreased. It was necessary to further analyse the changes of these substances during this period.

### 3.3. HS-GC-MS analysis

#### 3.3.1. Sum of peak areas of volatile components

To explore the volatile flavour characteristics of CF at different roasting stages and their relationship with processing, HS-GC-MS was performed to analyse the volatile substances in the samples. The summed peak areas of the volatile components in CF at different roasting times are shown in Supplementary Figure 3. With prolonged roasting, the total amount of volatile components in the samples initially increased and subsequently decreased, as consistent with the sensor response trends obtained using the E-nose. When the roasting time was ≤ 4 min and the sample surface temperature was < 100°C, the total amount of volatile components did not change significantly. However, when the surface temperature exceeded 100°C, the total amount of volatile components increased rapidly, indicating that the volatile components could only be completely released at > 100°C. Furthermore, the sum of the peak areas of the volatile components reached a maximum in S9 and S10 (roasting times of 16–18 min), implying that numerous substances increased in content or formed during this stage.

#### 3.3.2. Identification of volatile components in CF during roasting

**FIGURE 2:**
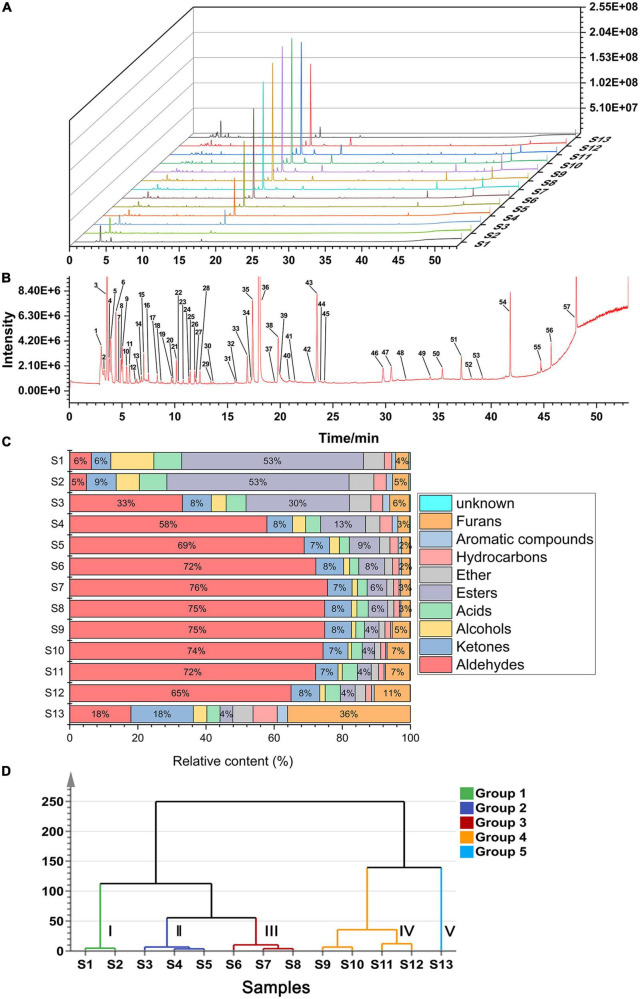
Analytical plots of CF samples in roasting process by HS-GC-MS. **(A)** Overlays of TICs of CF by HS-GC-MS; **(B)** typical TIC and distribution of volatile compounds in CF; **(C)** percentage chart of all kinds of volatile substances in CF; **(D)** HCA plot of CF by HS-GC-MS.

The total ion chromatogram (TIC) curves of the volatile components during roasting are shown in Figure 2A. Overall, 57 chromatographic peaks were detected and marked in Figure 2B. A total of 54 volatile compounds were identified using the MS and RI database. Based on this analysis, 14 common compounds were found in 13 samples, which were hexanal, furfural, acetone, 2,3-butanedione, 4-methyl-2-pentanone, ethanol, acetic acid, methyl formate, dimethyl ether, limonene, 1-methyl-4-(1-methylethylidene)-cyclohexene, toluene, *o*-cymene, and 2-methyl-furan. In addition, compared to the original sample (S1), 19 novel compounds were produced during roasting, of which 12 were produced at 4 min and then disappeared at 22 min, and the other 9 compounds disappeared. The volatile compounds are listed in Table 1 and the peak areas for the 13 samples are listed in Supplementary Table 2. Considering their chemical properties, the volatile compounds were divided into 9 categories that included 8 aldehydes, 14 ketones, 6 alcohols, 3 acids, 6 esters, 2 ethers, 7 hydrocarbons, 4 arenes, and 4 furans. The relative percentage accumulation diagram is shown in Figure 2C. Aldehydes, ketones, esters, and furans accounted for a large proportion of the volatile components, and significantly contributed to the CF flavour.

**TABLE 1:** Volatile compounds identified in CF during roasting by HS-GC-MS.

Peak No.	RT^a^	Compounds name	Formula	CAS	LRI^b^	MS match factor^c^
**Aldehydes (8)**						
8^d^	4.91	2-Methyl-butanal	C_5_H_10_O	96-17-3	869	892
16*	7.51	Hexanal	C_6_H_12_O	66-25-1	1039	898
31^d^	15.71	Nonanal	C_9_H_18_O	124-19-6	1335	813
36*	18.02	Furfural	C_5_H_4_O_2_	98-01-1	1394	941
40^f^	20.75	Benzaldehyde	C_7_H_6_O	100-52-7	1443	828
43^d^	23.49	5-Methyl-2-furancarboxaldehyde	C_6_H_6_O_2_	620-02-0	1490	915
55^e^	44.72	5-Acetoxymethyl-2-furaldehyde	C_8_H_8_O_4_	10551-58-3	2136	889
57^d^	48.08	5-Hydroxymethylfurfural	C_6_H_6_O_3_	67-47-0	2441	910
**Ketones (14)**						
5*	3.93	Acetone	C_3_H_6_O	67-64-1	781	948
7^d^	4.69	2-Butanone	C_4_H_8_O	78-93-3	849	900
11*	5.71	2,3-Butanedione	C_4_H_6_O_2_	431-03-8	930	952
12*	6.27	4-Methyl-2-pentanone	C_6_H_12_O	108-10-1	967	835
15^d^	7.06	2,3-Pentanedione	C_5_H_8_O_2_	600-14-6	1015	926
17^f^	8.34	3-Penten-2-one	C_5_H_8_O	625-33-2	1083	986
25^d^	11.46	Dihydro-2-methyl-3(2H)-furanone	C_5_H_8_O_2_	3188-00-9	1209	950
27^f^	11.99	Acetoin	C_4_H_8_O_2_	513-86-0	1225	972
30^f^	13.64	6-Methyl-5-hepten-2-one	C_8_H_14_O	110-93-0	1277	880
45^e^	24.22	4-Cyclopentene-1,3-dione	C_5_H_4_O_2_	930-60-9	1502	838
47^e^	30.53	5-Methyl-2(5H)-Furanone	C_5_H_6_O_2_	591-11-7	1590	921
49^e^	34.23	2(5H)-Furanone	C_4_H_4_O_2_	497-23-4	1673	876
54^e^	41.81	1-(2-Furanyl)-2-hydroxy-ethanone	C_6_H_6_O_3_	17678-19-2	1947	912
56^e^	45.69	2,3-Dihydro-3,5-dihydroxy-6-methyl-4H-pyran-4-one	C_6_H_8_O_4_	28564-83-2	2214	890
**Alcohols (6)**						
9*	5.02	Ethanol	C_2_H_6_O	64-17-5	879	956
13^f^	6.69	2-Methyl-3-buten-2-ol	C_5_H_10_O	115-18-4	994	848
20^d^	9.83	2-Methyl-1-butanol	C_5_H_12_O	137-32-6	1146	895
46^e^	29.76	2-Furanmethanol	C_5_H_6_O_2_	98-00-0	1579	868
48^e^	31.92	2-Methyl-5-(1-methylethenyl)-, (1α,2β,5α)-cyclohexanol	C_10_H_18_O	38049-26-2	1617	777
53^e^	39.18	3-acetoxy-7,8-Epoxylanostan-11-ol	C_32_H_54_O_4_	917486-30-7	1823	796
**Acids (3)**						
35*	17.38	Acetic acid	C_2_H_4_O_2_	64-19-7	1378	976
39^e^	19.97	Formic acid	CH_2_O_2_	64-18-6	1430	811
41^d^	21.40	Propanoic acid	C_3_H_6_O_2_	79-09-4	1454	849
**Esters (6)**						
3*	3.58	Methyl formate	C_2_H_4_O_2_	107-31-3	750	933
33^d^	16.87	5-Methyl-2(3H)-furanone	C_5_H_6_O_2_	591-12-8	1365	881
37^e^	19.46	Furfuryl formate	C_6_H_6_O_3_	13493-97-5	1421	818
42^d^	23.26	Pentanoic acid, 4- oxo-, methyl ester	C_6_H_10_O_3_	624-45-3	1486	833
44^d^	23.84	Methyl 2-furoate	C_6_H_6_O_3_	611-13-2	1496	885
51^d^	37.18	Dihydro-3-methylene-5-methyl-2-furanone	C_6_H_8_O_2_	62873-16-9	1756	920
**Ether (1)**						
1*	3.02	Dimethyl ether	C_2_H_6_O	115-10-6		927
**Hydrocarbons (7)**						
2^d^	3.33	1,4-Pentadiene	C_5_H_8_	591-93-5	727	938
19^f^	9.69	1-Methyl-4-(1-methylethyl)-1,3-cyclohexadiene	C_10_H_16_	554-61-0	1141	835
21*	10.15	Limonene	C_10_H_16_	138-86-3	1159	910
22^f^	10.34	β-Phellandrene	C_10_H_16_	555-10-2	1166	859
24^f^	11.33	γ-Terpinene	C_10_H_16_	99-85-4	1204	905
28*	12.39	1-Methyl-4-(1-methylethylidene)-cyclohexene	C_10_H_16_	586-62-9	1238	867
50^e^	35.38	1-Methyl-3-(1-methylethyl)-cyclohexene	C_10_H_18_	13828-31-4	1702	812
**Arenes (4)**						
14*	6.84	Toluene	C_7_H_8_	108-88-3	1003	861
18^d^	8.71	*p*-Xylene	C_8_H_10_	106-42-3	1102	901
26*	11.89	*o*-Cymene	C_10_H_14_	527-84-4	1222	906
34^f^	17.27	1-Methyl-4-(1-methylethenyl)-benzene	C_10_H_12_	1195-32-0	1375	892
**Furans (5)**						
4^d^	3.80	Furan	C_4_H_4_O	110-00-9	770	932
6*	4.41	2-Methyl-furan	C_5_H_6_O	534-22-5	824	933
10^d^	5.44	2,5-Dimethyl-furan	C_6_H_8_O	625-86-5	912	906
23^d^	10.80	2-(Methoxymethyl)-furan	C_6_H_8_O_2_	13679-46-4	1185	912
38^d^	19.81	1-(2-furanyl)-ethanone	C_6_H_6_O_2_	1192-62-7	1427	915
**Unknown (3)**						
29	13.43	Unknown			1271	
32	15.87	Unknown			1339	
52	38.15	Unknown			1785	

^a^RT: Retention time.

^b^LRI: Linear retention index on the HP-INNOWAX capillary column (Agilent Technologies), calculated *via* the C7-C30 n-alkanes.

^c^The maximum of MS match factor is 1,000.

^d^Compounds produced during roasting.

^e^Compounds produced at first and then disappeared during roasting.

^f^Compounds disappeared during roasting. *Common peaks.

To further examine the similarities and differences among the samples during different roasting stages, hierarchical cluster analysis (HCA) was performed using Ward’s clustering method. As shown in Figure 2D, when the distance is 50, the 13 samples can be divided into Group I (S1 and S2), Group II (S3, S4, and S5), Group III (S6, S7, and S8), Group IV (S9, S10, S11, and S12), and Group V (S13). The samples in Group I were abundant in esters, accounting for ≥ 50% of the total volatiles. In addition, the proportion of volatile compounds changed considerably in the Group V samples, where the esters reduced to 4%, while furans, aldehydes, and ketones accounted for 36, 18, and 18%, respectively. Furthermore, the samples in Groups II, III, and IV contained similar volatile substance compositions, where aldehydes accounted for the highest proportion and reached≥ 70% in Groups III and IV.

#### 3.3.3. Comprehensive analysis of volatile components

##### 3.3.3.1. Aldehydes

The 8 aldehydes detected showed high contents and significant changes, and were considered to be the main components that caused flavour changes during CF roasting. 2-methyl-butanal, nonanal, 5-methyl-2-furancarboxaldehyde, 5-acetoxymethyl-2-furaldehyde, and 5-hydroxymethylfurfural are the newly produced aldehydes in this roasting process. With increasing roasting time, the contents of hexanal and benzaldehyde decreased sharply, while the content of other aldehydes initially increased and subsequently decreased. It was reported that CF could promote digestion after roasting due to the increased content of 2-methyl-butanal, 5-hydroxymethylfurfural and furfural (25–27), and our experimental results are consistent with those reports. In addition, furfural and 5-hydroxymethylfurfural are typical thermal reaction products formed by the Maillard reaction (28). Meanwhile, the appearance of CF changed from red to black during roasting. In conclusion, the changes in colour and composition proved that the Maillard reaction was the main reaction during CF roasting. Previous research has shown that CF is rich in fatty acids such as linoleic acid and oleic acid, and hexanal and nonanal are typical oxidative volatile degradation products of these fatty acids. Therefore, it was speculated that aldehyde production during CF roasting was related to fatty acid oxidation and degradation (29). Furthermore, the 2-methyl-butanal and benzaldehyde produced during roasting were Strecker degradation reaction products (30). Because CF is rich in amino acids, it was speculated that significant production aldehydes during roasting was also related to the Strecker degradation reaction, a thermal reaction between amino acids and carbonyl compounds (31). Additionally, amino acids and fatty acids in CF are non-volatile, the changes in non-volatile components in CF samples during roasting must be further studied to confirm the contribution of Strecker degradation and fatty acid oxidation and degradation to overall flavour characteristics.

##### 3.3.3.2. Ketones

The proportion of ketones (14 compounds) was relatively stable during CF roasting and reached a maximum in S13 (roasting time of 24 min). Among the identified ketones, acetone and 1-(2-furanyl)-2-hydroxy-ethanone accounted for a relatively high proportion. Throughout roasting, the CF odour profile changed from fragrant to coke-like. The acetone content gradually increased, which contributed to the deterioration of flavour (32). Eight ketones were newly produced during roasting, while only three remained (2-butanone, 2,3-pentanedione and dihydro-2-methyl-3(2H)-furanone) after 24 min of roasting.

##### 3.3.3.3. Alcohols, acids, esters, and others

The newly produced alcohols during CF roasting are 2-methyl-1-butanol, 2-furanmethanol, and 2-methyl-5-(1-methylethenyl)-, (1α, 2β, 5α)-cyclohexanol. Among these, only the 2-methyl-1-butanol contents continued to increase. 2-Furanmethanol is often produced during the Maillard reaction (33). Acetic acid accounted for a relatively high proportion of acids, and its change trend was the same as that of most components during the roasting period. Acetic acid is thought to be produced by the secondary decomposition of hexanal during heating (34). The increased composition of acetic acid not only stimulates the secretory function of the oxyntic glands in the stomach, accelerating gastric breakdown and transformation (35), but also dilates blood vessels and reduces blood pressure (36, 37). Five of the six ester compounds in CF were newly produced compounds, including furfuryl formate, pentanoic acid, 4- oxo-, methyl ester, methyl 2-furoate, and dihydro-3-methylene-5-methyl-2-furanone. The decreased content of methyl formate, the most abundant ester compound, was likely related to its thermal decomposition (38).

Prolonged roasting times and increased CF temperature of promoted the production of other components such as 1,4-pentadiene, 1-methyl-3-(1-methylethyl)-cyclohexene, *p*-xylene, furan, 2,5-dimethyl-furan, 1-(2-furanyl)-ethanone, and 2-(methoxymethyl)-furan. The production of hydrocarbons, arenes, and furans in CF was likely promoted by chemical reactions including the Maillard reaction and protein degradation (39).

In conclusion, volatile components were developed through three pathways during CF roasting: Maillard reaction, Strecker degradation, and fatty acid oxidation and degradation.

### 3.4. Relative odour activity value analysis

The significance of the volatile compound contribution to the overall aroma depends on two factors, namely, their concentration and OT. ROAV represent the above two factors, and were used to assess the contribution of each volatile compound to the overall aromatic characteristics (40). To determine the volatiles that contributed significantly to CF odour during roasting, the ROAVs of all volatiles were calculated using OT literature value (41). Because 2,3-butanedione had the lowest OT and a high relative content, it was considered as the volatile component with the greatest contribution to flavour. Its ROAV was defined as 100 for comparison with other compounds. Generally, compounds with ROAV ≥ 1 were considered to be key aromatic compounds. Supplementary Table 3 shows 11 volatile compounds with ROAVs ≥ 1, namely, 2,3-butanedione, 2-methyl-butanal, 3-penten-2-one, hexanal, nonanal, limonene, 2,3-pentanedione, furfural, 5-methyl-2-furancarboxaldehyde, 1-methyl-4-(1-methylethenyl)-benzene, and 6-methyl-5-hepten-2-one (sorted by ROAV from large to small). These compounds were mainly aldehydes and ketones, which were inferred to be the main odour components in roasted CF. Generally, the sensory threshold of small-molecule aldehydes is lower and significantly contributes to the overall CF aroma. Based on the odour description of volatile components listed in Supplementary Table 3, aldehydes have aromatic characteristics including fruity, sour, and almond odours. Ketones have an important effect on fruit aroma. Its contribution to odour is less than that of aldehydes. The contribution of 2-methyl-butanal (ROAV 12.28–263.42) to the aroma was the second-greatest throughout CF roasting, although its content change was not significant. The ROAV of furfural showed a broad range from 0.28 to 12.27. Although the content of furfural varied the most, its contribution to aroma was not as high as that of 2-methyl-butanal. Furthermore, there were 5 components with ROAVs between 0 and 1, which are modifiable components of odour. Unfortunately, the OT values of 20 components have not been reported, requiring further studies to prove inference reliability.

### 3.5. Chemometric analysis

#### 3.5.1. PCA

Principal component analysis is a common analysis method for visualising sample differences, reducing data dimensionality while retaining most information by studying all the variable relationships simultaneously (42). The results obtained using the E-nose and GC-MS were statistically analysed by PCA to highlight the differences in volatile flavour components.

**FIGURE 3:**
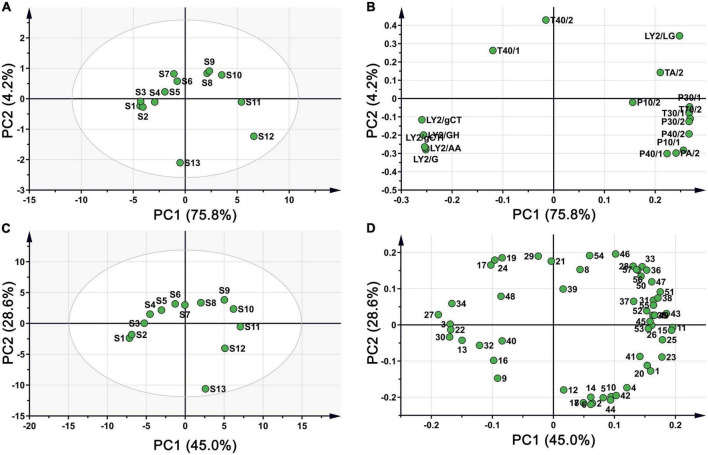
PCA plot of CF samples during roasting process. **(A)** Score plot based on E-nose; **(B)** loading plot based on E-nose; **(C)** score plot based on HS-GC-MS; **(D)** loading plot based on HS-GC-MS.

The score plot of the E-nose response is presented in Figure 3A, where higher contributions more significantly contribute toward the principal components (PCs) that reflect the original multi-index information. The first two principal components (75.8 and 4.2% for PC1 and PC2, respectively) explained 80% of the total variance, indicating that the two components contributed most to the characteristic information of the original samples after dimensionality reduction. The sample spatial regions showed a clockwise rotation with prolonged roasting time. S1, S2, and S3 were close in dimensionality, indicating that negligible changes in volatile flavour substances within 4 min. A similar aggregation in the PCA was observed for S8 and S9. With increasing roasting time, the distance between the samples gradually increased with large separation of S11, S12, and S13 from the previous samples. This indicated that roasting for 20 min at 150°C considerably impacted the volatile flavour substances in CF. Overall, the samples were distinguishable across PC1: S1–S7 and S13 had negative values on PC1, while S8–S12 occupied positive values on PC1. The loading plot in Figure 3B shows the influence of each sensor on sample differentiation.

The volatile components detected by HS-GC-MS were also analysed using PCA. From Figure 3C, the cumulative contribution rate of the two PCs exceeded 70%, and the spatial regions of the samples showed a clockwise rotation with prolonged roasting. The distance between samples at each time point gradually increased, similar to the PCA distribution of the E-nose data. These results further suggested that the E-nose and HS-GC-MS can be used to evaluate the quality of CF during processing, with consistent results across platforms. The loading plot in Figure 3D shows the influence of each compound on sample differentiation.

#### 3.5.2. Grey relational analysis between the E-nose and HS-GC-MS

**FIGURE 4:**
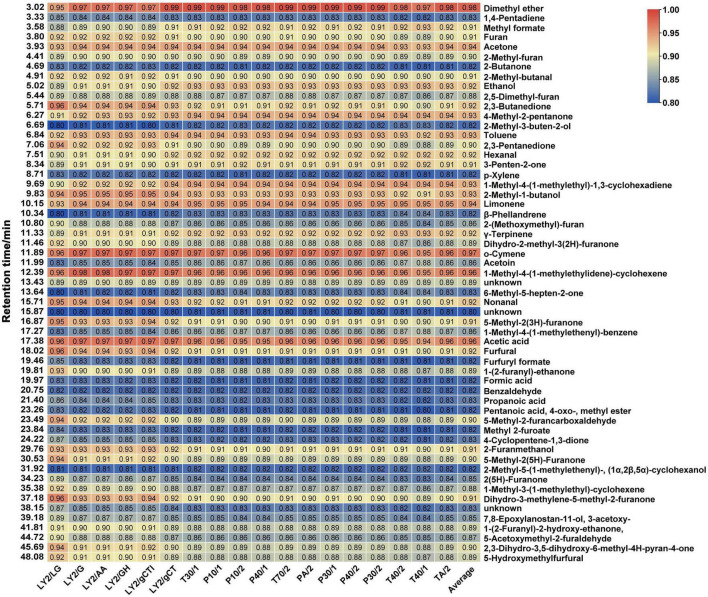
Heatmap of sensor response and volatile components based on grey relational analysis. GRA, a method to evaluate the correlation between two variables based on their change curves geometric shape similarity, is suitable for analyzing dynamically changing variables. Each row in the figure represents a volatile compound, and each column represents an E-nose sensor. The data values in the square represent the correlation degree. Red represents the correlation degree higher than 0.9 while blue represents the correlation degree lower than 0.9.

To further verify the correlation between the data obtained by the E-nose and GC-MS and explore the key components that cause odour changes during CF roasting, the grey relational method was used to calculate the correlation between each E-nose sensor response and volatile compound. The degree of correlation is shown in the two-colour gradient heatmap in Figure 4, where the *X*-axis represents sensors and the *Y*-axis represents volatile components. The last column represents the average of the correlation degree between each compound and sensor. In GRA, based on the criterion of the average correlation degree (*r* > 0.9) of 18 sensors (43), 25 known volatile components were screened, mainly aldehydes, ketones, and alcohols. They were dimethyl ether, *o*-cymene, 1-methyl-4-(1-methylethylidene)-cyclohexene, acetic acid, limonene, acetone, 1-methyl-4-(1-methylethyl)-1,3-cyclohexadiene, 2-methyl-1-butanol, toluene, 4-methyl-2-pentanone, nonanal, 2,3-butanedione, ethanol, γ-terpinene, furfural, hexanal, 5-methyl-2(3h)-furanone, dihydro-3-methylene-5-methyl-2-furanone, 2-furanmethanol, 3-penten-2-one, methyl formate, furan, 2-methyl-butanal, 2,3-pentanedione, and 5-methyl-2-furancarboxaldehyde (sorted by ROAV from large to small). By combining the ROAVs of the components in Supplementary Table 3, the key odour compounds were further screened (ROAV ≥ 1), which were limonene, 2,3-butanedione, nonanal, furfural, hexanal, 3-penten-2-one, 2-methyl-butanal, 2,3-pentanedione, and 5-methyl-2-furancarboxaldehyde. These compounds have special odour based on the odour description, and most of them have biological activities. For instance, limonene, nonanal, hexanal, and 2,3-pentanedione have antimicrobial activity (44–47). GC-MS and the E-nose are different odour judgment technologies that characterise odours by different indicative values and the correlative components are related to the odour of the food. Therefore, the 9 volatile components are identified as the main substances driving odour changes during CF roasting.

## 4. Conclusion

Herein, the E-nose and HS-GC-MS were used to analyse the changes in volatile flavour substances during CF roasting. The results obtained by the E-nose were consistent with those obtained by HS-GC-MS, indicating that both methods can be used to track and detect the odour changes in CF. During CF roasting, 54 volatile components were detected and identified by HS-GC-MS, mostly aldehydes, ketones, acids, esters, and furans, which are the main substances contributing to the CF flavour. It was confirmed that the Maillard reaction, Strecker degradation, and fatty acid oxidation and degradation were the three main reactions during roasting. The correlation between the E-nose response and volatile components was analysed by GRA, and 25 volatile components closely related to odour changes were identified. Finally, 9 volatile components (ROAV ≥ 1), mostly aldehydes and ketones, were identified as key substances driving odour changes during CF roasting. In summary, our research not only provides a template for seeking flavour markers in food processing, but also promotes the process of objectification and controllability of judgment of processing degree.

## Data availability statement

The raw data supporting the conclusions of this article will be made available by the authors, without undue reservation.

## Author contributions

CF performed the experiments and wrote the original draft. QX performed the GC-MS and E-nose data analysis. WJL carried out the mathematical analysis and validation. YX and LM applied the software and data visualization. WDL and TL collected the samples and edited the draft. LL and WY conceived the project and provided the guidance. FY conceived the project, provided the guidance, and edited the draft. All authors reviewed and approved the final version to be submitted.

## Footnotes

1
http://www.tbtools.com/

## References

[1] Augustin M Riley M Stockmann R Bennett L Kahl A Lockett T Role of food processing in food and nutrition security. *Trends Food Sci Technol.* (2016) 56:115–25. 10.1016/j.tifs.2016.08.005

[2] Dong W Hu R Long Y Li H Zhang Y Zhu K Comparative evaluation of the volatile profiles and taste properties of roasted coffee beans as affected by drying method and detected by electronic nose, electronic tongue, and HS-SPME-GC-MS. *Food Chem.* (2019) 272:723–31. 10.1016/j.foodchem.2018.08.068 30309604

[3] Sruthi N Premjit Y Pandiselvam R Kothakota A Ramesh S. An overview of conventional and emerging techniques of roasting: effect on food bioactive signatures. *Food Chem.* (2021) 348:129088. 10.1016/j.foodchem.2021.129088 33515948

[4] Hamzalioglu A Gokmen V. 5-Hydroxymethylfurfural accumulation plays a critical role on acrylamide formation in coffee during roasting as confirmed by multiresponse kinetic modelling. *Food Chem.* (2020) 318:126467. 10.1016/j.foodchem.2020.126467 32145542

[5] Zhu Y Dong J Jin J Liu J Zheng X Lu J Roasting process shaping the chemical profile of roasted green tea and the association with aroma features. *Food Chem.* (2021) 353:129428. 10.1016/j.foodchem.2021.129428 33714119

[6] Choi Y Yong S Lee M Park S Yun Y Park S Changes in volatile and non-volatile compounds of model kimchi through fermentation by lactic acid bacteria. *Lebensm Wiss Technol.* (2019) 105:118–26. 10.1016/j.lwt.2019.02.001

[7] Wang J Chen Q Hu K Zeng L Pan Y Huang H. Change in aromatic components of banana during the preparation process of juice and microcapsule powder. *Int J Food Sci Technol.* (2011) 46:1398–405. 10.1111/j.1365-2621.2011.02628.x

[8] Wang H Chen X Zhang J Wang X Shi W. Postmortem changes in the freshness and volatile compounds of grass carp (*Ctenopharyngodon idella*). *J Food Meas Charact.* (2020) 14:584–96. 10.1007/s11694-019-00337-8

[9] Siró I Kápolna E Kápolna B Lugasi A. Functional food, product development, marketing and consumer acceptance—A review. *Appetite.* (2008) 51:456–67. 10.1016/j.appet.2008.05.060 18582508

[10] Li Y Fei C Mao C Ji D Gong J Qin Y Physicochemical parameters combined flash GC e-nose and artificial neural network for quality and volatile characterization of vinegar with different brewing techniques. *Food Chem.* (2022) 374:131658. 10.1016/j.foodchem.2021.131658 34896949

[11] Tan J Xu J. Applications of electronic nose (e-nose) and electronic tongue (e-tongue) in food quality-related properties determination: a review. *Artif Intell Agric.* (2020) 4:104–15. 10.1016/j.aiia.2020.06.003

[12] Aparicio R Rocha S Delgadillo I Morales M. Detection of rancid defect in virgin olive oil by the electronic nose. *J Agric Food Chem.* (2000) 48:853–60. 10.1021/jf9814087 10725163

[13] Mohammadhosseini M Akbarzadeh A Flamini G. Profiling of compositions of essential oils and volatiles of *Salvia limbata* using traditional and advanced techniques and evaluation for biological activities of their extracts. *Chem Biodivers.* (2017) 14:e1600361. 10.1002/cbdv.201600361 28273408

[14] Nekoei M Mohammadhosseini M. Chemical composition of the essential oils and volatiles of *Salvia leriifolia* by Three different extraction methods prior to gas chromatographic-mass spectrometric determination: comparison of HD with SFME and HS-SPME. *J Essent Oil Bear Plants.* (2017) 20:410–25. 10.1080/0972060X.2017.1305918

[15] Xiang X Jin G Gouda M Jin Y Ma M. Characterization and classification of volatiles from different breeds of eggs by SPME-GC–MS and chemometrics. *Food Res Int.* (2019) 116:767–77. 10.1016/j.foodres.2018.09.010 30717006

[16] Shi H Zhang M Adhikari B. Advances of electronic nose and its application in fresh foods: a review. *Crit Rev Food Sci Nutr.* (2018) 58:2700–10. 10.1080/10408398.2017.1327419 28665685

[17] Zhou H Luo D GholamHosseini H Li Z He J. Identification of Chinese herbal medicines with electronic nose technology: applications and challenges. *Sensors.* (2017) 17:1073. 10.3390/s17051073 28486407 PMC5470463

[18] Li M Chen X Deng J Ouyang D Wang D Liang Y Effect of thermal processing on free and bound phenolic compounds and antioxidant activities of hawthorn. *Food Chem.* (2020) 332:127429. 10.1016/j.foodchem.2020.127429 32645678

[19] Li T Li S Dong Y Zhu R Liu Y. Antioxidant activity of penta-oligogalacturonide, isolated from haw pectin, suppresses triglyceride synthesis in mice fed with a high-fat diet. *Food Chem.* (2014) 145:335–41. 10.1016/j.foodchem.2013.08.036 24128486

[20] Zhang L Zhang L Xu J. Chemical composition, antibacterial activity and action mechanism of different extracts from hawthorn (*Crataegus pinnatifida Bge*.). *Sci Rep.* (2020) 10:8876. 10.1038/s41598-020-65802-7 32483369 PMC7264281

[21] Xue Q Wang Y Fei C Ren C Li W Li W Profiling and analysis of multiple constituents in *Crataegi Fructus* before and after processing by ultrahigh-performance liquid chromatography quadrupole time-of-flight mass spectrometry. *Rapid Commun Mass Spectrom.* (2021) 35:e9033. 10.1002/rcm.9033 33368723

[22] Fei C Ren C Wang Y Li L Li W Yin F Identification of the raw and processed *Crataegi Fructus* based on the electronic nose coupled with chemometric methods. *Sci Rep.* (2021) 11:1849. 10.1038/s41598-020-79717-w 33473146 PMC7817683

[23] Liu T Jiang H Li S. Analysis of volatile components in *Crataegus pinnatifida* and its processed products by HS-SPME-GC-MS. *Modern Food Sci Technol.* (2021) 37:250–5. 10.13982/j.mfst.1673-9078.2021.5.1026

[24] Shen Y Wu Y Wang Y Li L Li C Zhao Y Contribution of autochthonous microbiota succession to flavor formation during Chinese fermented mandarin fish (*Siniperca chuatsi*). *Food Chem.* (2021) 348:129107. 10.1016/j.foodchem.2021.129107 33515949

[25] Wang Y Lv M Wang T Sun J Wang Y Xia M Research on mechanism of charred hawthorn on digestive through modulating “brain-gut” axis and gut flora. *J Ethnopharmacol.* (2019) 245:112166. 10.1016/j.jep.2019.112166 31421184

[26] Xu Y. *Study on the “Coke Aroma” Material Basis That Promote Digestion and the Synergistic Mechanism of “Jiao Sanxian” After Charred.* Sichuan: Southwest Jiaotong University (2018).

[27] Zhou Y He F Yang Y Shi J Deng K Tang Y Research situation of Maillard reaction and its influence on research methods for processing and preparation process of Chinese materia medica. *Chin Traditi Herb Drugs.* (2014) 45:125–30. 10.7501/j.issn.0253-2670.2014.01.024

[28] Gong R Huo X Lei Z Liu C Li S Sun Y. Advances in effects and regulation of Maillard reaction on quality of Chinese materia medica. *Chin Tradit Herb Drugs.* (2019) 1:243–51. 10.7501/j.issn.0253-2670.2019.01.035

[29] Siegmund B Murkovic M. Changes in chemical composition of pumpkin seeds during the roasting process for production of pumpkin seed oil (Part 2: volatile compounds). *Food Chem.* (2004) 84:367–74.

[30] Pripis-Nicolau L de Revel G Bertrand A Maujean A. Formation of flavor components by the reaction of amino acid and carbonyl compounds in mild conditions. *J Agric Food Chem.* (2000) 48:3761–6. 10.1021/jf991024w 10995267

[31] Delgado R Hidalgo F Zamora R. Antagonism between lipid-derived reactive carbonyls and phenolic compounds in the Strecker degradation of amino acids. *Food Chem.* (2016) 194:1143–8. 10.1016/j.foodchem.2015.07.126 26471665

[32] Didzbalis J Ritter K Trail A Plog F. Identification of fruity/fermented odorants in high-temperature-cured roasted peanuts. *J Agric Food Chem.* (2004) 52:4828–33. 10.1021/jf0355250 15264922

[33] Ames J Guy R Kipping G. Effect of pH, temperature, and moisture on the formation of volatile compounds in glycine/glucose model systems. *J Agric Food Chem.* (2001) 49:4315–23. 10.1021/jf010198m 11559131

[34] Smuda M Glomb M. Fragmentation pathways during Maillard-induced carbohydrate degradation. *J Agric Food Chem.* (2013) 61:10198–208. 10.1021/jf305117s 23425499

[35] Foster N Lambert A. The effects of some organic acids on the secretion of gastric juice. *Proc Soc Exp Biol Med.* (1908) 5:108. 10.3181/00379727-5-64

[36] Li L He M Xiao H Liu X Wang K Zhang Y. Acetic acid influences BRL-3A cell lipid metabolism via the AMPK signalling pathway. *Cell Physiol Biochem.* (2018) 45:2021–30. 10.1159/000487980 29529605

[37] Kondo S Tayama K Tsukamoto Y Ikeda K Yamori Y. Antihypertensive effects of acetic acid and vinegar on spontaneously hypertensive rats. *Biosci Biotechnol Biochem.* (2001) 65:2690–4. 10.1271/bbb.65.2690 11826965

[38] Rhoades J. Coffee volatiles, analysis of the volatile constituents of coffee. *J Agric Food Chem.* (1960) 8:136–41. 10.1021/jf60108a019

[39] Su G Zheng L Cui C Yang B Ren J Zhao M. Characterization of antioxidant activity and volatile compounds of Maillard reaction products derived from different peptide fractions of peanut hydrolysate. *Food Res Int.* (2011) 44:3250–8. 10.1016/j.foodres.2011.09.009

[40] Zhang W Cao J Li Z Li Q Lai X Sun L HS-SPME and GC/MS volatile component analysis of Yinghong No. 9 dark tea during the pile fermentation process. *Food Chem.* (2021) 357:129654. 10.1016/j.foodchem.2021.129654 33866239

[41] van Gemert L. *Odor Thresholds Compilations of Odor Threshold Values in Air, Water and other Media.* Utrecht: Oliemans Punter & Partners BV (2011).

[42] Yang Y Wang B Fu Y Shi Y Chen F Guan H HS-GC-IMS with PCA to analyze volatile flavor compounds across different production stages of fermented soybean whey tofu. *Food Chem.* (2021) 346:128880. 10.1016/j.foodchem.2020.128880 33418415

[43] Han Y Cui S Geng Z Chu C Chen K Wang Y. Food quality and safety risk assessment using a novel HMM method based on GRA. *Food Control.* (2019) 105:180–9. 10.1016/j.foodcont.2019.05.039

[44] Zahi M Liang H Yuan Q. Improving the antimicrobial activity of d-limonene using a novel organogel-based nanoemulsion. *Food Control.* (2015) 50:554–9. 10.1016/j.foodcont.2014.10.001

[45] Zhang J Sun H Chen S Zeng L Wang T. Anti-fungal activity, mechanism studies on α-phellandrene and nonanal against *Penicillium cyclopium*. *Bot Stud.* (2017) 58:13. 10.1186/s40529-017-0168-8 28510196 PMC5430584

[46] Gardini F Lanciotti R Caccioni D Guerzoni M. Antifungal activity of hexanal as dependent on its vapor pressure. *J Agric Food Chem.* (1997) 45:4297–302. 10.1021/jf970347u

[47] Jay J Rivers G. Antimicrobial activity of some food flavoring compounds. *J Food Saf.* (1984) 6:129–39. 10.1111/j.1745-4565.1984.tb00609.x

