# Supplementary material for: Variations in volatile flavour compounds in Crataegi fructus roasting revealed by E-nose and HS-GC-MS
Source: Front Nutr. 2023 Jan 25;9:1035623. doi: 10.3389/fnut.2022.1035623 (PMC9905410; doi:10.3389/fnut.2022.1035623)
Supplement: Supplementary file 1 [file Data_Sheet_1.pdf]

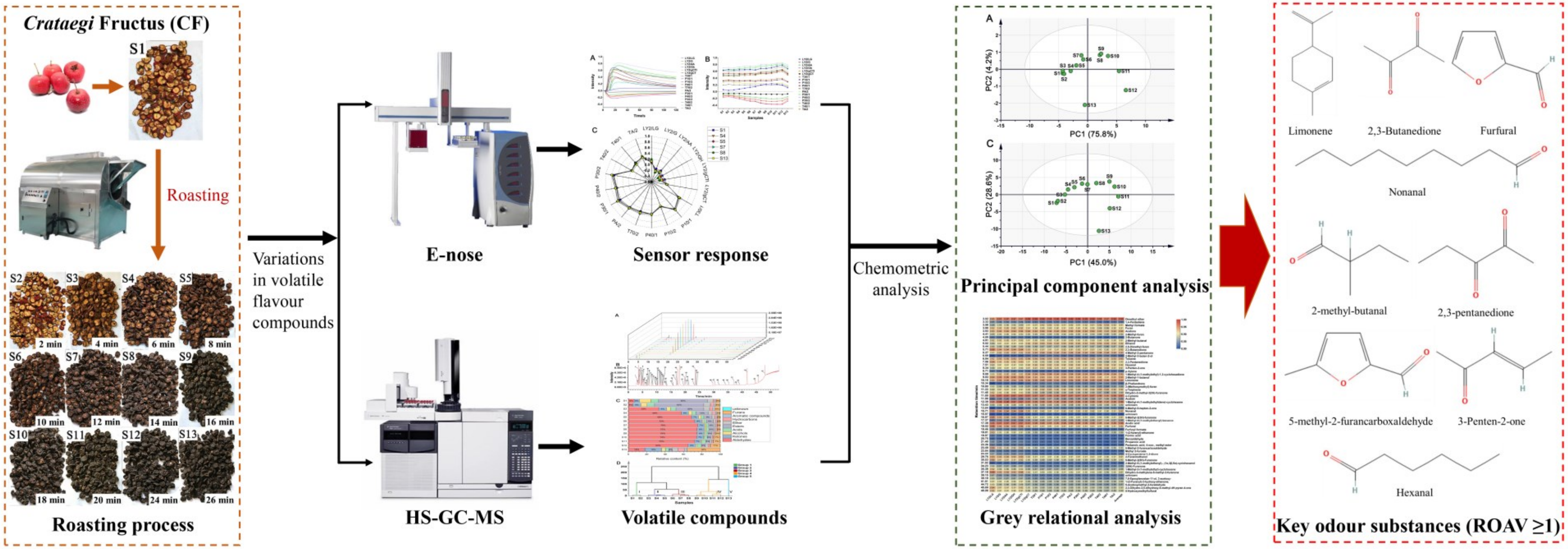


**Figure S1** The flowchart of the entire experiment.

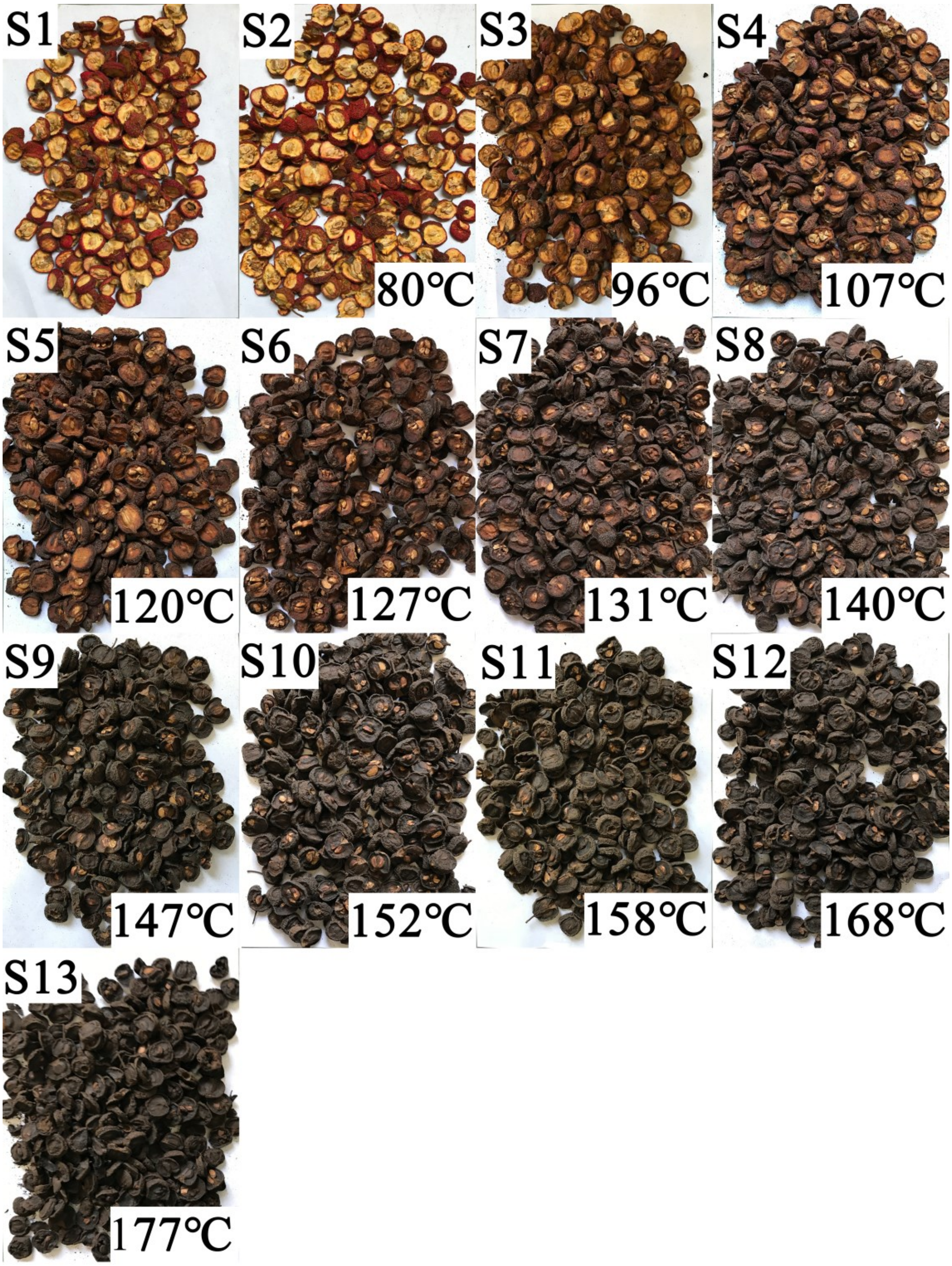


**Figure S2** CF samples during roasting process.
Sample number and roasting temperature are shown on top-left and lower-right corner, respectively.

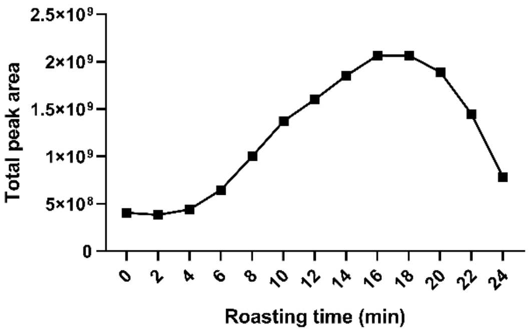


**Figure S3** The sum of peak areas of volatile components in CF at different roasting time.

**Table S1** Sensors and corresponding representative sensitive substances of Fox-4000.

| Sensors | Properties | Reference substances |
|---|---|---|
| LY2/LG | Sensitivity for gas with strong oxidation ability | Chlorine, fluorine, nitrogen oxides and sulfides |
| LY2/G | Sensitivity for toxic gas | Ammonia, amine compounds, carbon and oxygen compounds |
| LY2/AA | Sensitivity for organic compounds | Ammonia, ethanol and acetone |
| LY2/GH | Sensitivity for toxic gas | Ammonia and amine compounds |
| LY2/gCTl | Sensitivity for toxic gas | Hydrogen sulfide |
| LY2/gCT | Sensitivity for inflammable gas | Propane and butane |
| T30/1 | Sensitivity for organic compounds | Polar organic compound and hydrogen sulfide |
| P10/1 | Sensitivity for combustible gas | Carbon oxygen compound, ammonia and chlorine |
| P10/2 | Sensitivity for inflammable gas | Methane and ethane |
| P40/1 | Sensitivity for gas with strong oxidation ability | Chlorid and fluoride |
| T70/2 | Sensitivity for aromatic compounds | Toluene and xylene |
| PA/2 | Sensitivity for organic compounds and toxic gas | Ethanol, ammonia and amine compounds |
| P30/1 | Sensitivity for combustible gas and organic compounds | Carbon oxygen compound, combustion product, ammonia and ethanol |
| P40/2 | Sensitivity for gas with strong oxidation ability | Hydrogen sulfide, chlorine and fluoride |
| P30/2 | Sensitivity for organic compounds | Ethanol, combustion products, aldehydes and hydrogen sulfide |
| T40/2 | Sensitivity for gas with strong oxidation ability | Chlorid and fluoride |
| T40/1 | Sensitivity for gas with strong oxidation ability | Fluoride |
| TA/2 | Sensitivity for organic compounds | Ethanol |

**Table S2** Changes of peak area of volatile components in CF during roasting by HS-GC-MS.

| Peak No. | RT[a] | Compounds name | Average absolute peak area (×10$^6$) | | | | | | | | | | | | |
|---|---|---|---|---|---|---|---|---|---|---|---|---|---|---|---|
| | | | S1 | S2 | S3 | S4 | S5 | S6 | S7 | S8 | S9 | S10 | S11 | S12 | S13 |
| | | **Aldehydes (8)** | 25.56 | 18.81 | 145.30 | 373.64 | 689.07 | 991.46 | 1211.13 | 1385.49 | 1547.13 | 1534.61 | 1364.53 | 937.28 | 140.45 |
| 8 | 4.91 | 2-Methyl-butanal | - | - | 4.89 | 8.49 | 20.35 | 21.29 | 19.17 | 21.45 | 17.21 | 8.36 | 5.47 | 4.72 | 6.91 |
| 16* | 7.51 | Hexanal | 12.71 | 11.13 | 2.72 | 1.82 | 2.00 | 2.70 | 3.58 | 3.12 | 2.97 | 3.54 | 2.76 | 3.71 | 5.28 |
| 31 | 15.71 | Nonanal | - | - | - | - | 1.09 | 1.28 | 1.70 | 1.77 | 2.18 | 1.54 | 1.39 | 1.31 | 1.23 |
| 36* | 18.02 | Furfural | 9.58 | 7.68 | 133.35 | 349.57 | 631.89 | 891.62 | 1092.13 | 1233.84 | 1348.18 | 1338.51 | 1175.77 | 793.39 | 95.86 |
| 40 | 20.75 | Benzaldehyde | 3.27 | - | - | - | - | - | - | - | - | - | - | - | - |
| 43 | 23.49 | 5-Methyl-2-furancarboxaldehyde | - | - | 4.35 | 8.93 | 18.63 | 32.28 | 44.68 | 63.19 | 93.93 | 110.12 | 132.68 | 113.58 | 27.78 |
| 55 | 44.72 | 5-Acetoxymethyl-2-furaldehyde | - | - | - | - | - | 1.08 | 1.26 | 1.50 | 4.70 | 7.87 | 7.26 | 3.49 | - |
| 57 | 48.08 | 5-Hydroxymethylfurfural | - | - | - | 4.84 | 15.10 | 41.21 | 48.62 | 60.62 | 77.97 | 64.67 | 39.21 | 17.08 | 3.38 |
| | | **Ketones (14)** | 22.88 | 33.37 | 37.12 | 48.62 | 74.25 | 111.71 | 116.13 | 144.30 | 161.37 | 151.33 | 124.23 | 121.47 | 143.25 |
| 5* | 3.93 | Acetone | 15.07 | 22.13 | 15.98 | 16.25 | 22.32 | 23.29 | 21.72 | 25.09 | 27.70 | 26.72 | 27.80 | 51.83 | 97.07 |
| 7 | 4.69 | 2-Butanone | - | - | - | - | - | - | - | - | - | - | - | 3.91 | 11.90 |
| 11* | 5.71 | 2,3-Butanedione | 1.72 | 2.10 | 2.72 | 2.66 | 2.58 | 6.16 | 7.30 | 8.99 | 13.59 | 15.61 | 13.68 | 12.83 | 11.20 |
| 12* | 6.27 | 4-Methyl-2-pentanone | 0.57 | 2.01 | 1.22 | 1.03 | 1.07 | 2.06 | 1.14 | 1.21 | 1.31 | 0.66 | 1.03 | 1.48 | 4.65 |
| 15 | 7.06 | 2,3-Pentanedione | - | - | - | - | 2.90 | 7.64 | 7.35 | 11.81 | 15.87 | 22.89 | 22.14 | 17.47 | 14.12 |
| 17 | 8.34 | 3-Penten-2-one | 2.19 | 4.86 | 3.76 | 3.84 | 4.34 | 6.83 | 3.43 | 4.35 | 4.21 | 1.83 | - | - | - |
| 25 | 11.46 | Dihydro-2-methyl-3(2H)-furanone | - | - | - | - | - | 1.61 | 2.28 | 3.15 | 5.20 | 7.36 | 11.65 | 9.82 | 4.31 |
| 27 | 11.99 | Acetoin | 3.36 | 2.89 | 2.89 | 2.42 | 2.43 | 1.96 | 1.79 | - | - | - | - | - | - |
| 30 | 13.64 | 6-Methyl-5-hepten-2-one | 1.69 | 1.47 | 0.83 | 0.79 | 0.65 | - | - | - | - | - | - | - | - |
| 45 | 24.22 | 4-Cyclopentene-1,3-dione | - | - | - | - | - | - | - | - | 3.14 | 3.99 | 5.22 | 3.72 | - |
| 47 | 30.53 | 5-Methyl-2(5H)-Furanone | - | - | 1.45 | 1.53 | 4.29 | 7.56 | 10.23 | 13.05 | 19.79 | 24.92 | 19.59 | 8.24 | - |
| 49 | 34.23 | 2(5H)-Furanone | - | - | - | - | - | - | - | 2.95 | 2.72 | 3.77 | 4.66 | 4.20 | - |

| | | | | | | | | | | | | | | | |
|---|---|---|---|---|---|---|---|---|---|---|---|---|---|---|---|
| 54 | 41.81 | 1-(2-Furanyl)-2-hydroxy-ethanone | - | - | 8.26 | 18.81 | 30.66 | 48.28 | 54.21 | 63.71 | 53.02 | 35.13 | 10.66 | 4.20 | - |
| 56 | 45.69 | 2,3-Dihydro-3,5-dihydroxy-6-methyl-4H-pyran-4-one | - | - | - | 1.31 | 3.02 | 6.30 | 6.69 | 9.99 | 14.81 | 8.44 | 7.81 | 3.77 | - |
| | | **Alcohols (6)** | 50.90 | 26.06 | 19.12 | 24.64 | 29.28 | 24.37 | 24.84 | 31.62 | 28.39 | 23.92 | 28.11 | 27.33 | 31.08 |
| 9* | 5.02 | Ethanol | 47.43 | 19.89 | 10.83 | 13.10 | 16.92 | 6.58 | 8.11 | 11.89 | 7.69 | 5.77 | 11.77 | 15.96 | 27.42 |
| 13 | 6.69 | 2-Methyl-3-buten-2-ol | 3.47 | 6.17 | 2.69 | 1.79 | - | - | - | - | - | - | - | - | - |
| 20 | 9.83 | 2-Methyl-1-butanol | - | - | - | 1.05 | 1.17 | 1.27 | 1.23 | 1.40 | 1.65 | 1.90 | 2.02 | 2.44 | 3.66 |
| 46 | 29.76 | 2-Furanmethanol | - | - | 4.10 | 6.38 | 9.39 | 13.82 | 15.50 | 17.23 | 17.38 | 13.73 | 9.87 | 4.74 | - |
| 48 | 31.92 | 2-Methyl-5-(1-methylethenyl)-, (1$\alpha$,2$\beta$,5$\alpha$)-cyclohexanol | - | - | 1.50 | 2.32 | 1.80 | 2.70 | - | - | - | - | - | - | - |
| 53 | 39.18 | 3-acetoxy-7,8-Epoxylanostan-11-ol | - | - | - | - | - | - | - | 1.11 | 1.67 | 2.52 | 4.45 | 4.19 | - |
| | | **Acids (3)** | 32.78 | 31.05 | 25.57 | 28.58 | 29.08 | 36.71 | 45.94 | 61.76 | 52.73 | 65.01 | 83.56 | 64.41 | 30.28 |
| 35* | 17.38 | Acetic acid | 32.78 | 31.05 | 25.57 | 28.58 | 29.08 | 31.95 | 33.36 | 44.86 | 52.73 | 62.13 | 77.87 | 60.67 | 28.33 |
| 39 | 19.97 | Formic acid | - | - | - | - | - | 4.76 | 12.58 | 16.90 | - | - | - | - | - |
| 41 | 21.40 | Propanoic acid | - | - | - | - | - | - | - | - | - | 2.88 | 5.68 | 3.74 | 1.95 |
| | | **Esters (6)** | 214.68 | 204.82 | 133.16 | 85.10 | 89.10 | 104.52 | 92.32 | 104.38 | 86.04 | 72.23 | 72.49 | 58.17 | 29.32 |
| 3* | 3.58 | Methyl formate | 214.68 | 204.82 | 127.20 | 75.67 | 73.86 | 84.10 | 65.51 | 74.23 | 45.70 | 27.57 | 26.76 | 29.55 | 18.24 |
| 33 | 16.87 | 5-Methyl-2(3H)-furanone | - | - | 3.06 | 5.36 | 9.68 | 12.67 | 15.29 | 18.00 | 21.57 | 23.41 | 16.26 | 8.23 | 1.18 |
| 37 | 19.46 | Furfuryl formate | - | - | - | - | - | - | - | - | 2.81 | 1.78 | 2.50 | - | - |
| 42 | 23.26 | Pentanoic acid, 4-oxo-, methyl ester | - | - | - | - | - | - | - | - | - | - | 1.58 | 1.59 | 2.14 |
| 44 | 23.84 | Methyl 2-furoate | - | - | - | - | - | - | - | - | - | - | 3.32 | 3.62 | 6.16 |
| 51 | 37.18 | Dihydro-3-methylene-5-methyl-2-furanone | - | - | 2.90 | 4.07 | 5.56 | 7.75 | 11.52 | 12.15 | 15.96 | 19.48 | 22.08 | 15.19 | 1.61 |
| | | **Ether (1)** | 24.74 | 27.91 | 27.75 | 27.20 | 30.34 | 31.63 | 31.69 | 31.75 | 34.28 | 34.61 | 37.26 | 41.04 | 43.53 |
| 1* | 3.02 | Dimethyl ether | 24.74 | 27.91 | 27.75 | 27.20 | 30.34 | 31.63 | 31.69 | 31.75 | 34.28 | 34.61 | 37.26 | 41.04 | 43.53 |
| | | **Hydrocarbons (7)** | 8.76 | 13.94 | 15.31 | 23.27 | 24.19 | 26.17 | 25.61 | 30.02 | 33.91 | 29.35 | 30.51 | 25.24 | 55.67 |
| 2 | 3.33 | 1,4-Pentadiene | - | - | - | - | - | - | - | - | - | - | 10.29 | 14.22 | 48.73 |
| 19 | 9.69 | 1-Methyl-4-(1-methylethyl)-1,3-cyclohexadiene | 2.00 | 2.03 | 2.22 | 4.66 | 3.65 | 2.97 | 2.48 | 2.44 | 2.51 | 2.19 | 1.71 | - | - |

| | | | | | | | | | | | | | | | |
|---|---|---|---|---|---|---|---|---|---|---|---|---|---|---|---|
| 21* | 10.15 | Limonene | 2.08 | 5.52 | 6.39 | 8.91 | 9.59 | 10.72 | 8.36 | 9.15 | 9.22 | 6.64 | 4.54 | 2.96 | 4.85 |
| 22 | 10.34 | *β*-Phellandrene | 1.10 | 2.08 | 1.38 | 1.25 | 1.11 | - | - | - | - | - | - | - | - |
| 24 | 11.33 | *γ*-Terpinene | 1.52 | 2.51 | 2.65 | 4.14 | 3.66 | 3.51 | 2.35 | 2.27 | 3.22 | 1.19 | 1.26 | - | - |
| 28* | 12.39 | 1-Methyl-4-(1-methylethylidene)-cyclohexene | 2.06 | 1.81 | 2.67 | 4.32 | 4.97 | 5.08 | 5.09 | 4.99 | 6.04 | 5.21 | 5.23 | 5.07 | 2.10 |
| 50 | 35.38 | 1-Methyl-3-(1-methylethyl)-cyclohexene | - | - | - | - | 1.21 | 3.89 | 7.34 | 11.17 | 12.92 | 14.12 | 7.48 | 2.98 | - |
| | | **Arenes (4)** | 4.51 | 7.08 | 8.88 | 10.48 | 9.58 | 9.48 | 7.66 | 8.44 | 9.54 | 8.46 | 7.48 | 11.56 | 23.53 |
| 14* | 6.84 | Toluene | 1.34 | 2.37 | 2.12 | 1.89 | 1.67 | 2.42 | 1.67 | 2.71 | 2.44 | 3.22 | 2.20 | 4.24 | 12.18 |
| 18 | 8.71 | *p*-Xylene | - | - | - | - | - | - | - | - | - | - | - | 1.64 | 4.81 |
| 26* | 11.89 | *o*-Cymene | 1.19 | 2.40 | 3.00 | 4.68 | 4.71 | 4.40 | 4.15 | 5.74 | 7.10 | 5.24 | 5.28 | 5.67 | 6.54 |
| 34 | 17.27 | 1-Methyl-4-(1-methylethenyl)-benzene | 1.99 | 2.31 | 3.77 | 3.91 | 3.20 | 2.66 | 1.84 | - | - | - | - | - | - |
| | | **Furans (5)** | 15.66 | 18.30 | 25.21 | 22.32 | 24.57 | 34.26 | 44.45 | 51.58 | 109.48 | 139.68 | 139.24 | 154.99 | 285.02 |
| 4 | 3.80 | Furan | - | - | 2.72 | 3.08 | 5.20 | 6.89 | 9.19 | 10.47 | 15.15 | 16.62 | 16.31 | 26.65 | 54.02 |
| 6* | 4.41 | 2-Methyl-furan | 15.66 | 18.30 | 19.58 | 13.97 | 9.90 | 8.75 | 9.05 | 8.84 | 18.68 | 27.02 | 34.29 | 67.14 | 184.92 |
| 10 | 5.44 | 2,5-Dimethyl-furan | - | - | - | - | - | 2.59 | 2.31 | 2.98 | 5.46 | 6.54 | 9.42 | 14.19 | 38.93 |
| 23 | 10.80 | 2-(Methoxymethyl)-furan | - | - | - | - | - | - | - | 1.27 | 2.34 | 2.82 | 3.21 | 3.19 | 2.83 |
| 38 | 19.81 | 1-(2-furanyl)-ethanone | - | - | 2.90 | 5.27 | 9.46 | 16.02 | 23.90 | 28.02 | 67.86 | 86.69 | 76.01 | 43.83 | 4.32 |
| | | **unknown (3)** | 1.88 | 1.80 | 1.52 | 1.72 | 1.99 | 2.12 | 1.69 | 1.65 | 3.68 | 4.84 | 3.00 | 1.39 | - |
| 29 | 13.43 | unknown | - | - | 1.52 | 1.72 | 1.99 | 2.12 | 1.69 | 1.65 | 1.40 | 1.20 | - | - | - |
| 32 | 15.87 | unknown | 1.88 | 1.80 | - | - | - | - | - | - | - | - | - | - | - |
| 52 | 38.15 | unknown | - | - | - | - | - | - | - | - | 2.28 | 3.64 | 3.00 | 1.39 | - |

[a] RT = Retention time.

* Common peaks; "-", not detectable.

**Table S3** Odour contribution of volatile components in CF samples during roasting process

| Peak No. | RT[a] | Compounds name | Odour thresholds ($mg\cdot kg^{-1}$)[b] | Relative odour activity values (ROAV, %) | | | | | | | | | | | | | Odour description[c] |
|---|---|---|---|---|---|---|---|---|---|---|---|---|---|---|---|---|---|
| | | | | S1 | S2 | S3 | S4 | S5 | S6 | S7 | S8 | S9 | S10 | S11 | S12 | S13 | |
| | | Aldehydes (8) | | | | | | | | | | | | | | | |
| 8* | 4.91 | 2-Methyl-butanal | 0.003 | - | - | 59.85 | 106.60 | 263.42 | 115.09 | 87.50 | 79.54 | 42.21 | 17.86 | 13.33 | 12.28 | 20.58 | Almond, Apple, Burnt, Choking, Cocoa, Coffee, Fermented, Fruity, Green, Iodoform, Malty, Musty, Nutty, Powerful, Sickly, Sour |
| 16* | 7.51 | Hexanal | 0.0045 | 164.39 | 117.67 | 22.16 | 15.19 | 17.29 | 9.73 | 10.89 | 7.71 | 4.86 | 5.04 | 4.48 | 6.42 | 10.48 | Acorn, Aldehydic, Fatty, Fishy, Fresh, Fruity, Grassy, Green, Herbaceous, Leafy, Sharp, Strong, Sweaty, Tallowy, vinous |
| 31* | 15.71 | Nonanal | 0.001 | - | - | - | - | 42.36 | 20.72 | 23.26 | 19.74 | 16.07 | 9.88 | 10.14 | 10.22 | 11.03 | Aldehydic, Chlorine, Citrus, Fatty, Floral, Fresh, Fruity, Gaseous, Gravy, Green, Lavender, Melon, Orange, Orange peel, Orris, Peely, pungent (slightly), Rose, Soapy, Sweet, Tallowy, Waxy |
| 36* | 18.02 | Furfural | 2 | 0.28 | 0.18 | 2.45 | 6.58 | 12.27 | 7.23 | 7.48 | 6.86 | 4.96 | 4.29 | 4.30 | 3.09 | 0.43 | Almond, Baked, benzaldehyde, Bread, Fragrant, Sweet, Woody |
| 40 | 20.75 | Benzaldehyde | 0.35 | 0.54 | - | - | - | - | - | - | - | - | - | - | - | - | Almond, Bitter, Bitter almond, Burnt sugar, Cherry, Fruity, Oil, Sharp, Strong, Sweet, Woody |
| 43* | 23.49 | 5-Methyl-2-furancarboxaldehyde | 0.5 | - | - | 0.32 | 0.67 | 1.45 | 1.05 | 1.22 | 1.41 | 1.38 | 1.41 | 1.94 | 1.77 | 0.50 | Acidic, Almond, Burnt sugar, Caramelized, Coffee, Maple, Spicy |
| 55 | 44.72 | 5-Acetoxymethyl-2-furaldehyde | NA | | | | | | | | | | | | | | NA |
| 57 | 48.08 | 5-Hydroxymethylfurfural | 1000 | - | - | - | <0.1 | <0.1 | <0.1 | <0.1 | <0.1 | <0.1 | <0.1 | <0.1 | <0.1 | <0.1 | Caramelized, Cardboard, Chamomile flowers, |

| | | | | | | | | | | | | | | | | | |
|---|---|---|---|---|---|---|---|---|---|---|---|---|---|---|---|---|---|
| | | | | | | | | | | | | | | | | | Fatty, Musty, Waxy |
| | | Ketones (14) | | | | | | | | | | | | | | | |
| 5 | 3.93 | Acetone | 120 | ＜0.1 | ＜0.1 | ＜0.1 | ＜0.1 | ＜0.1 | ＜0.1 | ＜0.1 | ＜0.1 | ＜0.1 | ＜0.1 | ＜0.1 | ＜0.1 | ＜0.1 | Apple, characteristic, Fruity, Glue, Pear, Solvent, Sweet, Violet |
| 7 | 4.69 | 2-Butanone | 17 | - | - | - | - | - | - | - | - | - | - | - | ＜0.1 | ＜0.1 | Acetone, Butter, Cheese, Chemical, Chocolate, Etheral, Fragrant, Fruity, Gaseous, Pleasant, Pungent, Sharp, Sweet |
| 11* | 5.71 | 2,3-Butanedione | 0.001 | 100 | 100 | 100 | 100 | 100 | 100 | 100 | 100 | 100 | 100 | 100 | 100 | 100 | Butter, Caramelized, Chlorine, Creamy, Fruity, Pineapple, Pungent, Spirit, Strong, Sweet |
| 12 | 6.27 | 4-Methyl-2-pentanone | 0.24 | 0.14 | 0.40 | 0.19 | 0.16 | 0.17 | 0.14 | ＜0.1 | ＜0.1 | ＜0.1 | ＜0.1 | ＜0.1 | ＜0.1 | 0.17 | Dairy, Faint, Fruity, Green, Ketonic, Petroleum, Sharp, Solvent |
| 15* | 7.06 | 2,3-Pentanedione | 0.02 | - | - | - | - | 5.63 | 6.20 | 5.03 | 6.57 | 5.84 | 7.33 | 8.09 | 6.81 | 6.31 | Almond, Apple, Burnt, Butter, Butterscotch, Caramelized, Cheese, Creamy, Creamy, diacetyl, Fresh, Fruity, grain, Malty, Nutty, Oily, Pungent, sickly, Sweet |
| 17* | 8.34 | 3-Penten-2-one | 0.0015 | 84.90 | 154.23 | 92.12 | 96.39 | 112.26 | 73.86 | 31.27 | 32.23 | 20.65 | 7.81 | - | - | - | Acetone, Fishy, Fruity, Phenolic |
| 25 | 11.46 | Dihydro-2-methyl-3(2H)-furanone | NA | | | | | | | | | | | | | | Bread, Coffee, Nutty, Solvent, Sweet |
| 27 | 11.99 | Acetoin | 0.8 | 0.24 | 0.17 | 0.13 | 0.11 | 0.12 | ＜0.1 | ＜0.1 | - | - | - | - | - | - | Butter, Coffee, Creamy, Dairy, Fatty, Milky, Sweet, Woody |
| 30* | 13.64 | 6-Methyl-5-hepten-2-one | 0.05 | 1.97 | 1.40 | 0.61 | 0.59 | 0.51 | - | - | - | - | - | - | - | - | Apple, Blackcurrant, Citrus, Earthy, Fatty, fruit (boiled), fruity (sweet), Green, Mushroom, Musty, Pepper, Rubber, Vinyl, Woody |
| 45 | 24.22 | 4-Cyclopentene-1,3-dione | NA | | | | | | | | | | | | | | NA |

| | | | | | | | | | | | | | | | | | |
|---|---|---|---|---|---|---|---|---|---|---|---|---|---|---|---|---|---|
| 47 | 30.53 | 5-Methyl-2(5H)-Furanone | NA | | | | | | | | | | | | | | Spice |
| 49 | 34.23 | 2(5H)-Furanone | NA | | | | | | | | | | | | | | Butter |
| 54 | 41.81 | 1-(2-Furanyl)-2-hydroxy-ethanone | NA | | | | | | | | | | | | | | NA |
| 56 | 45.69 | 2,3-Dihydro-3,5-dihydroxy-6-methyl-4H-pyran-4-one | 35 | - | - | - | ＜0.1 | ＜0.1 | ＜0.1 | ＜0.1 | ＜0.1 | ＜0.1 | ＜0.1 | ＜0.1 | ＜0.1 | - | NA |
| | | Alcohols (6) | | | | | | | | | | | | | | | |
| 9 | 5.02 | Ethanol | 16 | 0.17 | ＜0.1 | ＜0.1 | ＜0.1 | ＜0.1 | ＜0.1 | ＜0.1 | ＜0.1 | ＜0.1 | ＜0.1 | ＜0.1 | ＜0.1 | ＜0.1 | Alcoholic, Ethanol, Pungent, Strong, Sweet, Weak |
| 13 | 6.69 | 2-Methyl-3-buten-2-ol | 100 | ＜0.1 | ＜0.1 | ＜0.1 | ＜0.1 | - | - | - | - | - | - | - | - | - | Earthy, Fruity, Herbaceous, Oily, Sweet |
| 20 | 9.83 | 2-Methyl-1-butanol | 0.25 | - | - | - | 0.16 | 0.18 | ＜0.1 | ＜0.1 | ＜0.1 | ＜0.1 | ＜0.1 | ＜0.1 | ＜0.1 | 0.13 | Alcoholic, Balsamic, Banana, Butter, Fusel, Iodoform, Malty, Oil, Onion (ripe), Sweet, Vinous, Winey |
| 46 | 29.76 | 2-Furanmethanol | 1.9 | - | - | ＜0.1 | 0.13 | 0.19 | 0.12 | 0.11 | 0.10 | ＜0.1 | ＜0.1 | ＜0.1 | ＜0.1 | - | Burnt |
| 48 | 31.92 | 2-Methyl-5-(1-methylethenyl)-, (1$\alpha$,2$\beta$,5$\alpha$)-cyclohexanol | NA | | | | | | | | | | | | | | Alcoholic, Bread, Burnt sugar, Caramelized, Chemical, Coffee, Creamy, Faint, Fermented, Musty, Sweet, Weak |
| 53 | 39.18 | 3-acetoxy-7,8-Epoxylanostan-11-ol | NA | | | | | | | | | | | | | | NA |
| | | Acids (3) | | | | | | | | | | | | | | | |
| 35 | 17.38 | Acetic acid | 210 | ＜0.1 | ＜0.1 | ＜0.1 | ＜0.1 | ＜0.1 | ＜0.1 | ＜0.1 | ＜0.1 | ＜0.1 | ＜0.1 | ＜0.1 | ＜0.1 | ＜0.1 | Acetic, Acidic, Odorless, Pungent, Sharp, Sour, Vinegar |
| 39 | 19.97 | Formic acid | 1240 | - | - | - | - | - | ＜0.1 | ＜0.1 | ＜0.1 | - | - | - | - | - | Acidic, Pungent, Vinegar |
| 41 | 21.40 | Propanoic acid | 0.96 | - | - | - | - | - | - | - | - | - | ＜0.1 | ＜0.1 | ＜0.1 | ＜0.1 | Vinegar, Pungent, pungent (slightly), Rancid, Soy, Vinegar |
| | | Esters (6) | | | | | | | | | | | | | | | |
| 3 | 3.58 | Methyl formate | 190 | ＜0.1 | ＜0.1 | ＜0.1 | ＜0.1 | ＜0.1 | ＜0.1 | ＜0.1 | ＜0.1 | ＜0.1 | ＜0.1 | ＜0.1 | ＜0.1 | ＜0.1 | Agreeable, Fruity, Plum |

| | | | | | | | | | | | | | | | | | |
|---|---|---|---|---|---|---|---|---|---|---|---|---|---|---|---|---|---|
| 33 | 16.87 | 5-Methyl-2(3H)-furanone | NA | | | | | | | | | | | | | | Amber, Coumarin, Nutty, Solvent, Sweet, Tobacco, Tonka broadbean |
| 37 | 19.46 | Furfuryl formate | NA | | | | | | | | | | | | | | Etheral |
| 42 | 23.26 | Pentanoic acid, 4-oxo-, methyl ester | NA | | | | | | | | | | | | | | Caramelized |
| 44 | 23.84 | Methyl 2-furoate | NA | | | | | | | | | | | | | | Fruity, Fungal, Mushroom, Sweet, Tobacco |
| 51 | 37.18 | Dihydro-3-methylene-5-methyl-2-furanone | NA | | | | | | | | | | | | | | NA |
| | | Ether (1) | | | | | | | | | | | | | | | |
| 1 | 3.02 | Dimethyl ether | NA | | | | | | | | | | | | | | Etheral |
| | | Hydrocarbons (7) | | | | | | | | | | | | | | | NA |
| 2 | 3.33 | 1,4-Pentadiene | NA | | | | | | | | | | | | | | NA |
| 19 | 9.69 | 1-Methyl-4-(1-methylethyl)-1,3-cyclohexadiene | NA | | | | | | | | | | | | | | NA |
| 21* | 10.15 | Limonene | 0.01 | 12.14 | 26.24 | 23.47 | 33.54 | 37.24 | 17.40 | 11.45 | 10.18 | 6.79 | 4.25 | 3.32 | 2.31 | 4.33 | Lemon, Orange |
| 22 | 10.34 | *β*-Phellandrene | 0.5 | 0.13 | 0.20 | 0.10 | <0.1 | <0.1 | - | - | - | - | - | - | - | - | Fruity, Herbaceous, Minty, Pleasant, Terpenic, Turpentine |
| 24 | 11.33 | *γ*-Terpinene | 1 | <0.1 | 0.12 | 0.10 | 0.16 | 0.14 | <0.1 | <0.1 | <0.1 | <0.1 | <0.1 | <0.1 | - | - | Citrus, Etheral, Fruity, Gasoline, Herbaceous, Lemon, Oily, Sweet, Terpenic, Turpentine, Woody |
| 28 | 12.39 | 1-Methyl-4-(1-methylethylidene)-cyclohexene | 0.2 | 0.60 | 0.43 | 0.49 | 0.81 | 0.97 | 0.41 | 0.35 | 0.28 | 0.22 | 0.17 | 0.19 | 0.20 | <0.1 | Anisic (slightly), Citrus, Fresh, Fruity, Herbaceous, Pine, Plastic, Sweet, Woody |
| 50 | 35.38 | 1-Methyl-3-(1-methylethyl)-cyclohexene | NA | | | | | | | | | | | | | | NA |
| | | Arenes (4) | | | | | | | | | | | | | | | |
| 14 | 6.84 | Toluene | 0.527 | 0.15 | 0.21 | 0.15 | 0.14 | 0.12 | <0.1 | <0.1 | <0.1 | <0.1 | <0.1 | <0.1 | <0.1 | 0.21 | Caramelized, Etheral, Fruity, Paint, Pungent, |

| | | | | | | | | | | | | | | | | | |
|---|---|---|---|---|---|---|---|---|---|---|---|---|---|---|---|---|---|
| | | | | | | | | | | | | | | | | | Rubber, Solvent, Sweet, Synthetic |
| 18 | 8.71 | *p*-Xylene | 0.53 | - | - | - | - | - | - | - | - | - | - | - | <0.1 | <0.1 | Aromatic, Cold meat fat |
| 26 | 11.89 | *o*-Cymene | NA | | | | | | | | | | | | | | NA |
| 34* | 17.27 | 1-Methyl-4-(1-methylethenyl)-benzene | 0.085 | 1.36 | 1.29 | 1.63 | 1.73 | 1.46 | 0.51 | 0.30 | - | - | - | - | - | - | Citrus, Clove, Fruity, Phenolic, Pine, Spicy |
| | | Furans (5) | | | | | | | | | | | | | | | |
| 4 | 3.80 | Furan | 4.5 | - | - | <0.1 | <0.1 | <0.1 | <0.1 | <0.1 | <0.1 | <0.1 | <0.1 | <0.1 | <0.1 | 0.11 | Spicy |
| 6 | 4.41 | 2-Methyl-furan | 3.5 | 0.26 | 0.25 | 0.21 | 0.15 | 0.11 | <0.1 | <0.1 | <0.1 | <0.1 | <0.1 | <0.1 | 0.15 | 0.47 | Acetone, Burnt, Chocolate, gassy (sweet), Metallic, Musty, Solvent |
| 10 | 5.44 | 2,5-Dimethyl-furan | NA | | | | | | | | | | | | | | Chemical, Gravy, Meaty, Roast |
| 23 | 10.80 | 2-(Methoxymethyl)-furan | NA | | | | | | | | | | | | | | Coffee |
| 38 | 19.81 | 1-(2-furanyl)-ethanone | 10 | - | - | <0.1 | <0.1 | <0.1 | <0.1 | <0.1 | <0.1 | <0.1 | <0.1 | <0.1 | <0.1 | <0.1 | Almond, Balsamic, Cereal, Cinnamon, Cocoa, Coffee, Smoky, Sweet, Tobacco |
| | | unknown (3) | | | | | | | | | | | | | | | |
| 29 | 13.43 | unknown | NA | | | | | | | | | | | | | | |
| 32 | 15.87 | unknown | NA | | | | | | | | | | | | | | |
| 52 | 38.15 | unknown | NA | | | | | | | | | | | | | | |

[a] RT: retention time.

[b] Odour thresholds were referenced from a book named odour thresholds compilations of odour threshold values in air, water and other media (second enlarged and revised edition) .

[c] Odour descriptions according to: Flavornet (http://www.flavornet.org/flavornet.html); Bedoukian Research (http://www.bedoukian.com/); Sigma Aldrich SAFC, Flavors and Fragrances (http://www.safcglobal.com/safc-supply-solutions/en-us/home/flavors-and-fragrances.html) and The Good Scents Company (http://www.thegoodscentscompany.com/).

“NA”, not available. “-”, not detectable. “*”, components with ROAV ≥1.
